# Psychological distress, anxiety, family violence, suicidality, and wellbeing in New Zealand during the COVID-19 lockdown: A cross-sectional study

**DOI:** 10.1371/journal.pone.0241658

**Published:** 2020-11-04

**Authors:** Susanna Every-Palmer, Matthew Jenkins, Philip Gendall, Janet Hoek, Ben Beaglehole, Caroline Bell, Jonathan Williman, Charlene Rapsey, James Stanley

**Affiliations:** 1 Department of Psychological Medicine, University of Otago, Wellington, New Zealand; 2 Department of Public Health, University of Otago, Wellington, New Zealand; 3 Department of Psychological Medicine, University of Otago, Christchurch, New Zealand; 4 Department of Population Health, University of Otago, Christchurch, New Zealand; 5 Department of Psychological Medicine, University of Otago, Dunedin, New Zealand; University of the Witwatersrand, SOUTH AFRICA

## Abstract

New Zealand’s early response to the novel coronavirus pandemic included a strict lockdown which eliminated community transmission of COVID-19. However, this success was not without cost, both economic and social. In our study, we examined the psychological wellbeing of New Zealanders during the COVID-19 lockdown when restrictions reduced social contact, limited recreation opportunities, and resulted in job losses and financial insecurity. We conducted an online panel survey of a demographically representative sample of 2010 adult New Zealanders in April 2020. The survey contained three standardised measures–the Kessler Psychological Distress Scale (K10), the GAD-7, and the Well-Being Index (WHO-5)–as well as questions designed specifically to measure family violence, suicidal ideation, and alcohol consumption. It also included items assessing positive aspects of the lockdown. Thirty percent of respondents reported moderate to severe psychological distress (K10), 16% moderate to high levels of anxiety, and 39% low wellbeing; well above baseline measures. Poorer outcomes were seen among young people and those who had lost jobs or had less work, those with poor health status, and who had past diagnoses of mental illness. Suicidal ideation was reported by 6%, with 2% reporting making plans for suicide and 2% reporting suicide attempts. Suicidality was highest in those aged 18–34. Just under 10% of participants had directly experienced some form of family harm over the lockdown period. However, not all consequences of the lockdown were negative, with 62% reporting ‘silver linings’, which included enjoying working from home, spending more time with family, and a quieter, less polluted environment. New Zealand’s lockdown successfully eliminated COVID-19 from the community, but our results show this achievement brought a significant psychological toll. Although much of the debate about lockdown measures has focused on their economic effects, our findings emphasise the need to pay equal attention to their effects on psychological wellbeing.

## Introduction

The first identified case of COVID-19 was confirmed in New Zealand (NZ) on 28 February 2020, with case numbers increasing exponentially through March. On 21 March, Prime Minister Jacinda Ardern announced a country-wide alert level system, with 1 being the lowest level and 4 the highest. NZ moved rapidly to level 4 ‘lockdown’ and national state of emergency on 25 March, with all schools and non-essential businesses being shut and non-essential workers required to remain at home. The level 4 lockdown lasted for 33 days, ending on 28 April, when the country shifted to less stringent public health measures.

The lockdown was part of a ‘go early, go hard’ COVID-19 elimination strategy articulated by the NZ government [1]. It was implemented ‘early’, at a time when infection and mortality rates were comparatively low [2]. The restrictions were also ‘hard’ by any standard: on the Oxford University COVID-19 stringency index (a composite measure based on nine national pandemic response indicators to the pandemic including school closures, workplace closures, and travel bans), NZ scored 96/100, the highest of any World Bank high-income country [3]. These stringent measures were successful. At the end of lockdown in late April 2020, the estimated viral reproductive rate (R_0_) was 0.48, with daily case numbers in single figures, falling to zero in the following weeks [2, 4]. This was then followed by 3 months over the vulnerable winter period with no evidence of COVID-19 in the community [5]. A smaller regional outbreak in August 2020 was controlled by similar but slightly less stringent restrictions to the earlier lockdown.

Yet, when the March lockdown was first implemented, it was not at all certain that the elimination goal would be achieved. The restrictions posed significant costs to the economic and mental wellbeing of New Zealanders. These included reduced family and social contact; limited recreation opportunities; job losses and financial insecurity; and inability to attend universities and schools. It was predictable that both the restrictions and the fear generated by the pandemic itself would have significant effects on population mental health [6].

Early in 2020, the World Health Organization voiced concerns about the effects COVID-19 might have on psychological wellbeing [6]. However, actual evidence of the psychological impacts of the pandemic is only starting to emerge, with a growing number of studies suggesting increased anxiety and depression during stay-at-home orders. An early Chinese study comparing Weibo social-media posts from almost 18,000 users before and after declaration of the COVID-19 epidemic reported increases in negative emotions (e.g., anxiety, depression and indignation), and decreases in positive emotions (e.g., Oxford happiness scores) and life satisfaction [7]. Another study found 54% of Chinese citizens rated the pandemic as having moderate to severe psychological impacts on them [8]. Essential workers, particularly health care professionals, were reported to be at increased risk of negative psychological effects [9]. Overall, studies from Europe, North America, South America, and Asia are consistently reporting increased rates of COVID-related psychological distress amongst the population, particularly symptoms of anxiety and depression [e.g., 7–13].

Isolation can exacerbate pre-existing depression and anxiety [14], with potential deleterious effects on those with lived experience of mental distress. Reduced social connection has been shown to be a risk factor for suicidal behaviour [15]. Speculation about COVID-19-related suicide risk has to date largely been limited to opinion pieces [16] and news stories linking specific deaths by suicide to COVID-19-related experiences [17]. The British Royal College of Psychiatrists publicly commented on a six-fold increase in suicide attempts amongst the elderly in some areas [18].

There has been anecdotal reporting on psychosocial distress and its correlates in NZ but little empirical data. Mental health-related calls to Lifeline (a free helpline for those in distress) were reported by the media to have increased by 40% within the first weeks of the lockdown [19]. Increases in intimate partner violence were described as rising in parallel with the lockdown [20], with the police and Women’s Refuge reporting surges in family harm related calls [21]. Various groups expressed concerns about the availability and potential abuse of alcohol, which was available as an ‘essential item’ during the lockdown [22].

The length and severity of NZ’s strict lockdown measures have been vigorously debated, but most attention has been directed at their economic consequences. Discussion of their implications for psychological wellbeing have featured less prominently. This study was designed to address this information lacuna by investigating New Zealanders’ responses to the current pandemic lockdown and comparing the results with corresponding normative data.

The aims were to assess levels of psychological distress, anxiety, wellbeing, suicidal ideation, alcohol consumption, and family relationships using validated measures with benchmark comparisons where possible. A secondary aim was to identify specific populations (e.g., essential workers, those with underlying health conditions and the elderly) who might be more psychologically vulnerable to poor outcomes throughout the pandemic.

## Materials and methods

### Objectives

The specific objectives of our study were to determine:

1. The state of the NZ population’s wellbeing during the COVID-19 lockdown (stress, anxiety, depressive symptoms, alcohol consumption, family relationships, suicidal thinking etc).
2. How the lockdown impacted on specific populations (e.g., essential workers, those with underlying health conditions, and the elderly).
3. Whether there were any positive psychological consequences associated with the lockdown.

### Participants and recruitment methods

We recruited a large, demographically representative sample of adult New Zealanders aged between 18 and 90 years using a commercial survey platform, Dynata. Dynata has a New Zealand panel that currently stands at 300,000 active members (i.e., people who have participated in a survey in the last three months). Panel members are recruited through online methods such as Google AdWords campaigns, and via partners such as GrabOne and TradeMe. Respondents are incentivised by earning cash credits in online accounts.

This cross-sectional survey had a target of 2000 respondents in order to provide subgroups large enough to enable comparisons. The sample recruitment was designed to be representative of the age-sex distribution of the NZ population over 18 years, and with sufficient Māori and Pacific respondents to produce reliable estimates of ethnicity patterns, with target participation quotas by age, sex, and ethnicity.

**Fig 1:**
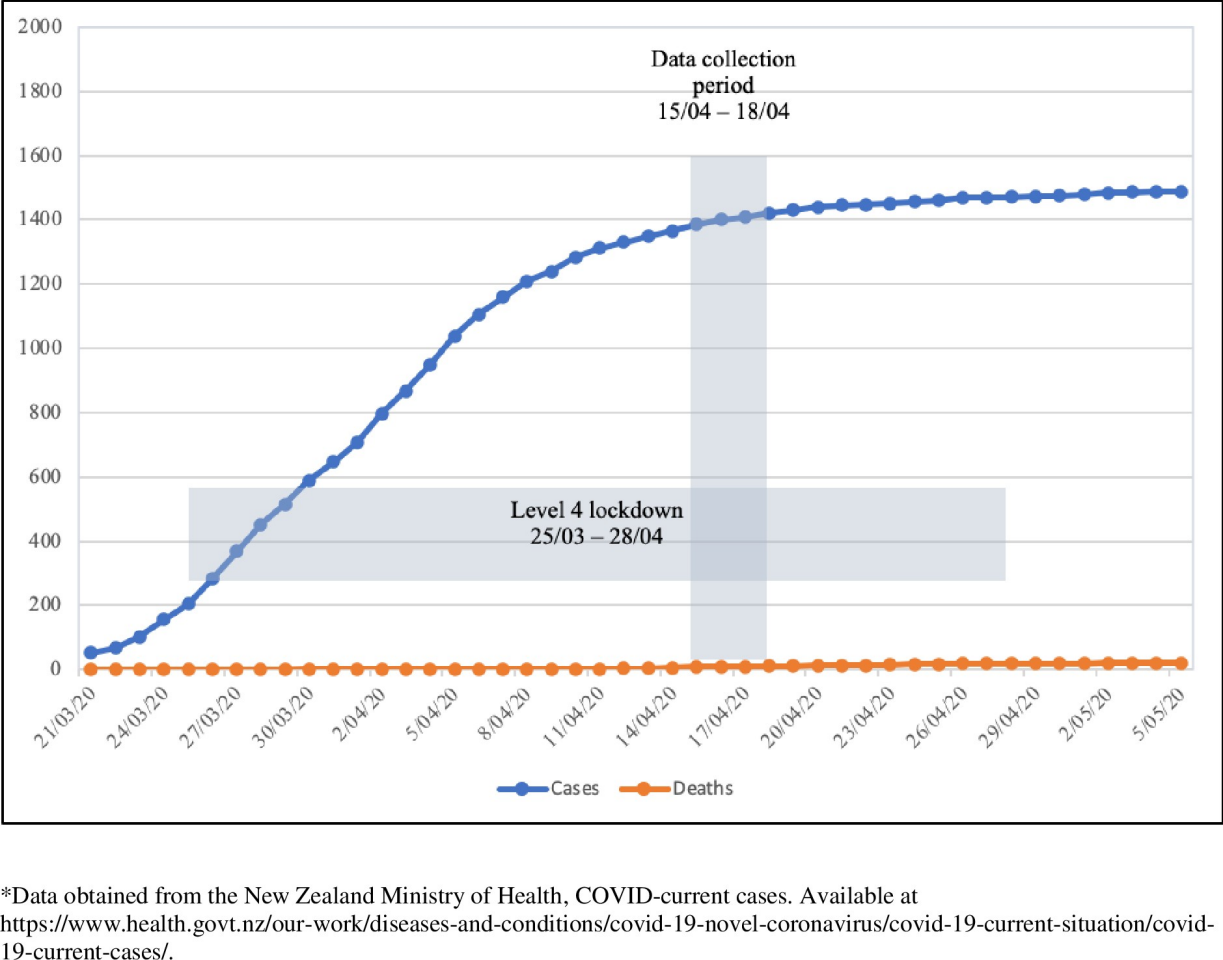
Data collection period, lockdown dates, and cases and deaths due to COVID-19 in New Zealand.

The survey was fielded between 15 and 18 April 2020, which corresponded with Alert Level 4 lockdown days 19 to 22. Between the start of the pandemic and the launch of our survey on 15 April there were 1366 cases of COVID-19 identified in NZ and nine deaths reported [5]. During the survey period, the number of cases rose by 56 and a further two deaths were reported. The relationship between data collection, the lockdown, and COVID-19 cases and deaths is shown in Fig 1.

The survey was fielded using the Qualtrics platform. It took approximately 15 minutes to complete (median 13.1, interquartile range 9.4–19.0). All participants were asked to read a participant information sheet and provide consent before they could proceed with the survey.

### Measures

The survey questions are available are a (S1 File). Demographic information collected included: age, gender (male, female, gender diverse), income, education, and ethnicity. Ethnicity was collected using the NZ Census question on ethnicity, which allows individuals to specify multiple ethnic groups. For analysis, individuals were prioritised into ethnic groups following standard guidelines [23].

Participants were also asked about their living circumstances, including questions pertaining to: who they lived with during the lockdown (family, friends, flatmates or tenants); how many people they lived with; level of satisfaction with their living situation; access to personal space, green space, internet and a computer; and ease and frequency of connection with family outside of their household. We inquired whether participants were ‘essential workers’–people required to continue working outside their home during the lockdown to provide necessities, maintain public health and safety and maintain key infrastructure [24].

The Kessler Psychological Distress Scale (K10) was the primary outcome measure [25]. This is a 10-item scale measuring non-specific symptoms of anxiety and depression over the previous four weeks. Scores are reported in a 0–40 range to align with reporting in the NZ Health Survey. People scoring 12 or higher have a high probability of meeting diagnostic criteria for anxiety or depressive disorders. We selected the K10 because it is a well-validated measure with good baseline data for the NZ population–it was an outcome measure in Te Rau Hinengaro: the NZ Mental Health Survey [26] and has been used in the NZ Health Survey since 2011 [27].

Symptoms of generalised anxiety disorder were measured using the Generalised Anxiety Disorder Assessment (GAD-7), which has been validated within large samples of patients in primary care [28, 29] and general population samples [30]. Respondents indicate how much they have been bothered by each of seven symptoms over the last two weeks, on a 4-point Likert scale ranging from ‘not at all’ to ‘nearly every day’. Scores range from 0–21 with a cut-off score of 10 and higher scores indicating higher anxiety.

The third key outcome was the World Health Organization Well-Being Index 5 (WHO-5), which is one of the most widely-used scales for assessing subjective psychological well-being [31]. The WHO-5 contains five positively phrased items, with respondents rating each statement for the last two weeks. Scores range from 0–25 with higher scores indicating higher subjective wellbeing. We were also interested in using the WHO-5 to characterise the group with excellent wellbeing during the lockdown restrictions. Again, there were good baseline population data for the WHO-5, with it having been used in NZ-wide population surveys since 2014 (e.g., NZ General Social Survey [32]).

Questions about family violence were adapted from standard questions asked in the New Zealand Crime and Victims of Crime Survey [33]. Suicidality was assessed using questions on suicidal ideation, suicide plans, and suicide attempts during the lockdown and the preceding 12 months. These questions were similar to those used by Te Rau Hinengaro: the NZ Mental Health Survey [34].

We assessed alcohol consumption by asking participants how many standard drinks they consumed on an average 7-day period before the lockdown and how many standard drinks they had consumed over the previous 7 days. We included a visual aid to help participants define a typical standard drink (for beer, wine and spirits).

Bearing in mind previous research that has described people’s experiences of ‘silver linings’–or benefit finding–during crises [e.g., 35, 36], we asked participants if the lockdown had produced any ‘silver linings’ for them personally or for NZ society in general. Those who answered ‘Yes’ to either question were asked to describe these benefits.

We designed the survey to ensure compatibility with mobile phone, tablet or computer access. We refined it using the ‘group mind’ process, which Sudman and Bradburn described as asking colleagues to review and rigorously critique a draft of the questionnaire [37], and made iterative improvements based on their comments. We then pre-tested the revised questionnaire on a small sample of the general public and further modified it to address respondents' feedback.

### Ethics approval

The study was approved by the University of Otago Human Ethics Committee (F20/003) and we undertook Māori consultation via the Ngāi Tahu Research Committee.

### Statistical analyses

Survey responses were weighted using a post-stratification weighting scheme to reflect the 2018 NZ Census adult (age 15+) population profile by age group, sex, and ethnicity (coded to Māori, Pacific, Asian, and European/Other). These post-stratification weights were checked for extreme weights that would indicate potential influence on estimates from participants where only a small number of participants represented a particular age/sex/ethnicity combination (all scaled weights < 4) [38].

Unweighted frequencies are reported to reflect absolute numbers of responses for a given question, and the available sample size is reported for sub-group and regression analyses. Unweighted percentages are presented for demographic variables, together with weighted percentages which describe the likely distribution of the demographic variables in the population (based on the sample data).

Weighted percentages for each outcome (unadjusted for additional covariates) are presented as percentages, with 95% confidence intervals (CIs). Unadjusted differences in outcomes by key sociodemographic variables (age, sex, ethnicity) are presented as odds ratios (ORs) with 95% confidence intervals (95% CI) derived from logistic regression models. Adjusted ORs are presented with mutual adjustment for age, sex, and ethnicity (i.e., a single logistic regression model with all three covariates included) as ORs with 95% CIs. For measures where the absolute numbers were low (e.g., suicidal thoughts and behaviours), these are not reported broken down by other demographic variables.

Additional analysis of health and wellbeing outcomes are reported according to pre-defined priority groups (e.g., living alone or with others; prior history of mental health diagnosis). Unadjusted and adjusted estimates for differences in outcomes by these factors are presented as ORs and 95% CIs (adjustment was for age, sex and ethnicity as above).

Data preparation, weight calculations and analysis were all conducted in R 3.6.1 (R Institute, Vienna, Austria). All complex-survey weighted estimates were calculated using the survey package [39].

## Results

In total, there were 2416 survey responses. The non-completion rate, defined as those who opened and started but did not complete the survey before the cut-off time, was 16.8% (*n* = 406), producing a cleaned achieved sample of 2010 cases.

Table 1 shows the demographic profile for survey participants; 408 participants (20.3%, unweighted) reported Māori ethnicity and 115 (5.7%) Pacific ethnicity. The 35 respondents who reported both Māori and Pacific ethnicity were included as Māori following the prioritisation procedure [23]. The median age of respondents was 45 years. There were slightly more female participants than males. Only six people reported being gender diverse, with this group being too small to analyse separately.

**Table 1:** Sample demographic characteristics.

Variable	Unweighted	Unweighted	Weighted
	(n)	(%)	(%)
**Total**	2010	100	100
**Gender**			
Male	941	46.8	48.9
Female	1063	52.9	50.8
Gender diverse	6	0.3	0.3
**Age (years)**			
18–24	269	13.4	16.4
25–34	407	20.2	17.5
35–44	349	17.4	15.5
45–54	327	16.3	16.7
55–64	304	15.1	14.9
65–74	239	11.9	12.9
75+	115	5.7	6.0
**Ethnicity (prioritised)**			
Māori	408	20.3	13.9
Pacific	115	5.7	5.9
Asian	256	12.7	14.6
European/Other ethnicity	1231	61.2	65.6
**Living circumstances**			
Living alone	276	13.7	14.2
Living with others	1734	86.3	85.8
**Happiness with bubble**			
Dissatisfied or neutral	374	18.6	18.6
Satisfied or very satisfied	1636	81.4	81.4
**Employment status**			
Currently an employee	1022	50.8	50.9
Self-employed	112	5.6	5.7
Business owner	31	1.5	1.5
Not currently in workforce	451	22.4	20.8
Retired	329	16.4	17.4
Never had job	65	3.2	3.6
**Type of work**			
Non-essential worker	772	38.4	38.7
Essential worker	393	19.6	19.4
Not working	845	42.0	41.9
**Health vulnerabilities**^$^			
Health vulnerabilities	482	24.5	22.6
No vulnerabilities	1483	75.5	75.3
**Self-rated health**			
Poor or fair health	450	22.4	21.5
Excellent, very good or good	1560	77.6	78.5
**History of mental illness**^%^			
Past history of mental illness	375	18.7	18.2
No past history of mental illness	1591	79.2	81.8

^$^ Responses missing for *n* = 45 respondents.

^%^ Responses missing for *n* = 44 respondents.

Most respondents (86.3% unweighted) spent the lockdown living with others, with 81.0% being happy with their lockdown living circumstances. The majority (77.6%) rated their health as good. Almost a quarter (23.0%) had pre-existing conditions or circumstances that made them vulnerable to coronavirus (e.g., being pregnant, immunocompromised). About one-fifth of participants (19.4% unweighted) were essential workers, with 113 employed in healthcare, 25 in law enforcement, 23 in other emergency services, and 232 as providers of essential goods or services (e.g., food supply, fuel, waste removal, internet, financial support, transport [24]).

Very few respondents (n = 36, 2.0% unweighted) believed they may have been infected with COVID-19. Nine people (0.3%) had returned positive serological tests. More than half of respondents (54%) reported spending over an hour a day looking at information related to COVID-19 in the media.

## Psychological distress

Just under one-third of participants (30.3%, 95% CI 28.3, 32.4) scored above the cut-off for moderate to severe psychological distress (K10 > 12). There was a strong gradient with prevalence of distress decreasing with age (see Table 2); almost half of younger adults scored highly on the K10 (47.3% of 18–24 year olds) compared to less than one in ten adults aged 65 years and older. These differences persisted following adjustment for covariates.

**Table 2:** Psychological distress: Those scoring in the categories of moderate or high psychosocial distress.

	Unweighted *n* with K10>12/*n*	Weighted % psychological distress		Adjusted OR psychological distress		
		Weighted % with K10>12	Weighted	Adjusted OR	Adjusted OR	p-value
			95% CI		95% CI	
**Total**	629/2010	30.3	(28.2, 32.4)	n/a		n/a
**Gender**^$^						
Male	264	28.2	(25.3, 31.3)	1.00 (ref)	Reference	0.026
Female	360	31.9	(29.1, 34.9)	1.27	(1.03, 1.57)	
**Age (years)**						
18–24	133	47.3	(41.1, 53.6)	1.46	(1.04, 2.05)	<0.001
25–34	191	47.1	(42.2, 52.0)	1.52	(1.12, 2.06)	
35–44	137	37.5	(32.3, 42.9)	1	(Reference)	
45–54	88	26.1	(21.5, 31.3)	0.57	(0.41, 0.80)	
55–64	48	15.8	(11.9, 20.7)	0.3	(0.20, 0.45)	
65–74	24	10.2	(6.8, 15.0)	0.18	(0.11, 0.29)	
75+	8	7.7	(3.9, 14.8)	0.13	(0.06, 0.27)	
**Ethnicity**						
Māori	153	38.1	(33.2, 43.2)	1.11	(0.84, 1.45)	0.027
Pacific	46	38.1	(28.9, 48.2)	1.09	(0.68, 1.73)	
Asian	80	28.4	(23.1, 34.3)	0.65	(0.47, 0.89)	
European/other	350	28.3	(25.8, 31.0)	1	(Reference)	
**Living circumstances**						
Living alone	79	28.6	(23.4, 34.5)	1.39	(1.02, 1.90)	0.037
Living with others	550	30.5	(28.3, 32.8)	1	(Reference)	
**Satisfactions with other members of household during lockdown**						
Satisfied or very satisfied	429	25.3	(23.2, 27.5)	1	(Reference)	<0.001
Dissatisfied or neutral	200	52.5	(47.0, 57.9)	3.55	(2.72, 4.66)	
**Work status**						
Non-essential	235	30.5	(27.3, 34.0)	1	(Reference)	0.076
Essential worker	135	33.5	(28.8, 38.5)	1.3	(0.97, 1.74)	
**Work Loss**						
Lost or less work	81	42	(34.7, 49.6)	1.75	(1.21, 2.52)	0.003
Not lost or less work	334	30.7	(27.9, 33.6)	1	(Reference)	
**Health vulnerabilities**						
Yes	167	33.7	(29.5, 38.2)	1.85	(1.42, 2.40)	<0.001
No vulnerabilities	441	28.7	(26.3, 31.1)	1	(Reference)	
**Self-rated health**						
Poor or fair health	211	46	(41.2, 50.9)	2.66	(2.07, 3.42)	<0.001
Excellent, very good or good	418	25.9	(23.7, 28.2)	1	(Reference)	
**History of mental illness**						
Past history	196	52.9	(47.6, 58.1)	2.9	(2.23, 3.78)	<0.001
No history	409	24.7	(22.5, 26.9)	1	(Reference)	

**Key** CI: confidence interval, K10: Kessler Psychological Distress Scale; *n*: sample size, OR: odds ratio.

^$^ Respondents identifying as gender diverse are not reported here.

Note: A total of 11 participants (0.5%) were missing K10 scores.

Substantially increased rates of distress were seen among those who reported having lost their jobs or experienced a reduction in work as a result of the pandemic, those who had potential vulnerabilities to COVID-19 (such as being pregnant or immunosuppressed) or identified their health status as poor, and those who had a past diagnosis of a mental illness. Females and those who lived alone had slightly increased adjusted odds of psychological distress.

There were no significant or substantial differences in rates of psychological distress between people identifying as Māori, Pacific Peoples, or NZ European, but those with Asian ethnicity reported lower rates of distress (analyses adjusted for age and sex profile).

### Anxiety and wellbeing

For anxiety, about one in six participants (15.6%, 95% CI 14.0, 17.3) reported moderate to high levels of anxiety (score of 10 or higher on the GAD-7). Low wellbeing was reported by 38.2% (95% CI 36.0, 40.4) of participants (WHO-5 score < 13), and 8.7% (95% CI 7.5, 10.1) reported excellent wellbeing (WHO-5 > 21).

Anxiety and wellbeing results are reported in Table 3 according to key sociodemographic characteristics. For both measures, patterning by age was similar to the current K10 results, with younger adults faring worse. There was no difference in prevalence between male and females, and for the WHO-5, Asian respondents had more favourable outcomes.

**Table 3:** Rates of anxiety and low wellbeing (measured by the GAD-7 and WHO-5).

**High anxiety score**	**Unweighted n**	**Weighted estimates**		**Adjusted OR**	**p-value**
**GAD-7>10**				**95% CI**	
		**%**	**(95% CI)**		
**Total**	322	15.6	(14.0, 17.3)		
**Gender**					
Male	145	15.5	(13.3, 18.1)	1.00 (Reference)	0.865
Female	172	15.2	(13.2, 17.6)	1.02 (0.79, 1.33)	
**Age (years)**					
18–24	85	30.3	(24.9, 36.2)	1.97 (1.33, 2.93)	<0.001
25–34	104	25.6	(21.5, 30.2)	1.64 (1.14, 2.38)	
35–44	64	17.3	(13.6, 21.8)	1.00 (Reference)	
45–54	36	10.5	(7.6, 14.4)	0.55 (0.34, 0.86)	
55–64	19	5.9	(3.7, 9.4)	0.3 (0.16, 0.52)	
65–74	9	3.6	(1.9, 6.9)	0.18 (0.08, 0.35)	
75+	5	5	(2.1, 11.5)	0.25 (0.09, 0.59)	
**Ethnicity**					
Māori	172	13.8	(12.0, 15.9)	1.24 (0.89, 1.71)	0.346
Pacific	81	21.7	(17.6, 26.4)	1.03 (0.61, 1.67)	
Asian	24	19.3	(12.8, 27.9)	0.8 (0.56, 1.22)	
European /Other ethnicity	45	16.2	(12.1, 21.3)	1.00 (Reference)	
**WHO-5 < 13**	**Unweighted**	**Weighted estimates**		**Adjusted OR**	**p-value**
**Low wellbeing score**	**(n)**	**%**	**(95% CI)**	**95% CI**	
**Total**	776	38.2	(36.0, 40.4)		
**Gender**					
Male	349	37.4	(34.2, 40.7)	1.00 (Reference)	0.346
Female	423	38.8	(35.8, 41.9)	1.1 (0.90, 1.33)	
**Age (years)**					
15–24	131	50	(43.8, 56.3)	1.05 (0.75, 1.47)	<0.001
25–34	198	48.5	(43.5, 53.4)	1.03 (0.76, 1.38)	
35–44	169	48.6	(43.1, 54.1)	1.00 (Reference)	
45–54	131	39.8	(34.5, 45.4)	0.67 (0.49, 0.93)	
55–64	81	25.3	(20.5, 30.7)	0.34 (0.24, 0.48)	
65–74	46	19	(14.3, 24.6)	0.23 (0.15, 0.34)	
**Ethnicity**					
Māori	161	39.8	(34.9, 45.0)	0.8 (0.62, 1.04)	0.009
Pacific	49	40.5	(31.2, 50.6)	0.79 (0.50, 1.24)	
Asian	97	34.8	(29.1, 41.1)	0.61 (0.46, 0.82)	
European /Other ethnicity	469	38.4	(35.7, 41.2)	1 (Reference)	

**Key** CI: confidence interval; GAD-7: Generalised Anxiety Disorder Assessment; *n*: sample size, OR: odds ratio; WHO-5: World Health Organization Well-Being Index 5.

Note: a total of 2 participants were missing the GAD-7 score; and 10 participants were missing their WHO-5 score.

### Psychological distress in those with past histories of mental illness

In our sample, 375 people (18.2%, 95% CI 16.5, 20.0) reported having previously been diagnosed with a mental health condition by a doctor or psychologist. Of these, many had more than one diagnosis, with 80.2% reporting having been diagnosed with a depressive disorder, 52.6% anxiety disorder, 5.8% personality disorder, 7.6% bipolar disorder, 5.7% an alcohol and drug disorder, 3. 9% a psychotic disorder, and 11.4% another disorder.

Over half of those with past mental health diagnoses were experiencing moderate or severe psychological distress (52.9%, 95% CI 47.6, 58.1). About one third (36.1%, 95% CI 31.2, 41.3) thought their mental health had been worse than usual during the lockdown, just under half thought it was the same as usual (46.1%, 95% CI 41.1, 51.6), and about one in six reported it was better than usual (17.5%, 95% CI 13.9, 21.8).

### Suicidal thoughts and behaviour

Suicidal ideation during lockdown was reported by 6.1% of participants (95% CI 5.1, 7.3) with 2.1% (95% CI 1.5, 2.9) reporting making plans for suicide and 2.1% also reporting a suicide attempt (95% CI 1.5, 2.9). Suicidality was highest in those age 18–34. For most of those experiencing suicidal thoughts, these were not new thoughts– 83.0% of that group reported having experienced similar ideation in the 12 months prior to lockdown.

### Alcohol intake

Since the beginning of the lockdown, about three in five participants (59.1%, 95% CI 56.9, 61.4) reported that their alcohol consumption had not changed, with about one-fifth reporting increased consumption (22.0%, 95% CI 20.2, 23.9), and another fifth reporting a decrease in consumption (18.9%, 95% CI 17.1, 20.7).

### Living circumstances and relationships

Many respondents appreciated having more time with their family and frequently cited this outcome as one of the pandemic’s ‘silver linings.’ In terms of immediate family relationships, more people reported their family relationships were better (24.5%, 95% CI 22.6, 26.6) or the same as usual (63.5%, 95% CI 61.2, 65.6) during the lockdown than worse than usual (12.0%, 95% CI 10.6, 13.6).

Most respondents (73.3%, 95% CI 71.2, 75.3) found it easy to maintain contact with friends and family members outside their home during the lockdown, while 11.6% (95% CI 10.2, 13.2) found it difficult to maintain these relationships. A small proportion (1.8%, 95% CI 1.3, 2.6) had not tried to maintain contact with friends and family members.

Despite many people reporting positive experiences at home, a significant minority described difficult living situations during the lockdown. The most commonly reported difficulties included loneliness all or most of the time (11.6%, 95% CI 10.2, 13.1) or some/a little of the time (49.8%, 95% CI 47.6, 52.1); and/or poor relationships with the other occupants of their household (bad/very bad: 4.6%, 95% CI 3.7, 5.7; neither well nor badly 17.7%, 95% CI 15.9, 19.7).

### Family harm

Almost one in ten participants (9.1%, 95% CI 7.9, 10.5) had directly experienced some form of family harm over the lockdown period, including sexual assault, physical assault, or harassment and threatening behaviour. Fewer people (3.9%, 95% CI 3.1, 4.9) reported witnessing family harm in which they were not the victim. There were 39 participants who said they preferred not to answer the family harm questions and 12 who gave no response (total 2.5%).

### Silver linings

The majority of participants (61.5%, 95% CI 59.3, 63.7) identified positive aspects of the lockdown, either for themselves personally (44.0%, 95% CI 41.7, 46.3) and/or for society (37.6%, 95% CI 35.4, 39.8). Common themes were that the lockdown was conducive to more family time, work flexibility and to social cohesion. People reported taking the opportunity to pause, reflect, consider priorities, recreate healthy habits, and they appreciated the environmental benefits brought by reduced travel.

## Discussion

### Key findings and comparison with benchmark data

While around two-thirds of participants appeared to be coping well with the pandemic lockdown, about one-third reported moderate or high psychosocial distress, rates well above baseline measures from past population surveys (NZ Health Survey 2018/19: 8.2% of total adult population, with rates higher in young people, females and Māori) [27]. Similarly, nearly 40% of participants reported experiencing poor wellbeing on the WHO-5, compared to 25% in a recent national level survey (NZ General Social Survey 2018/9 [32]).

**Fig 2:**
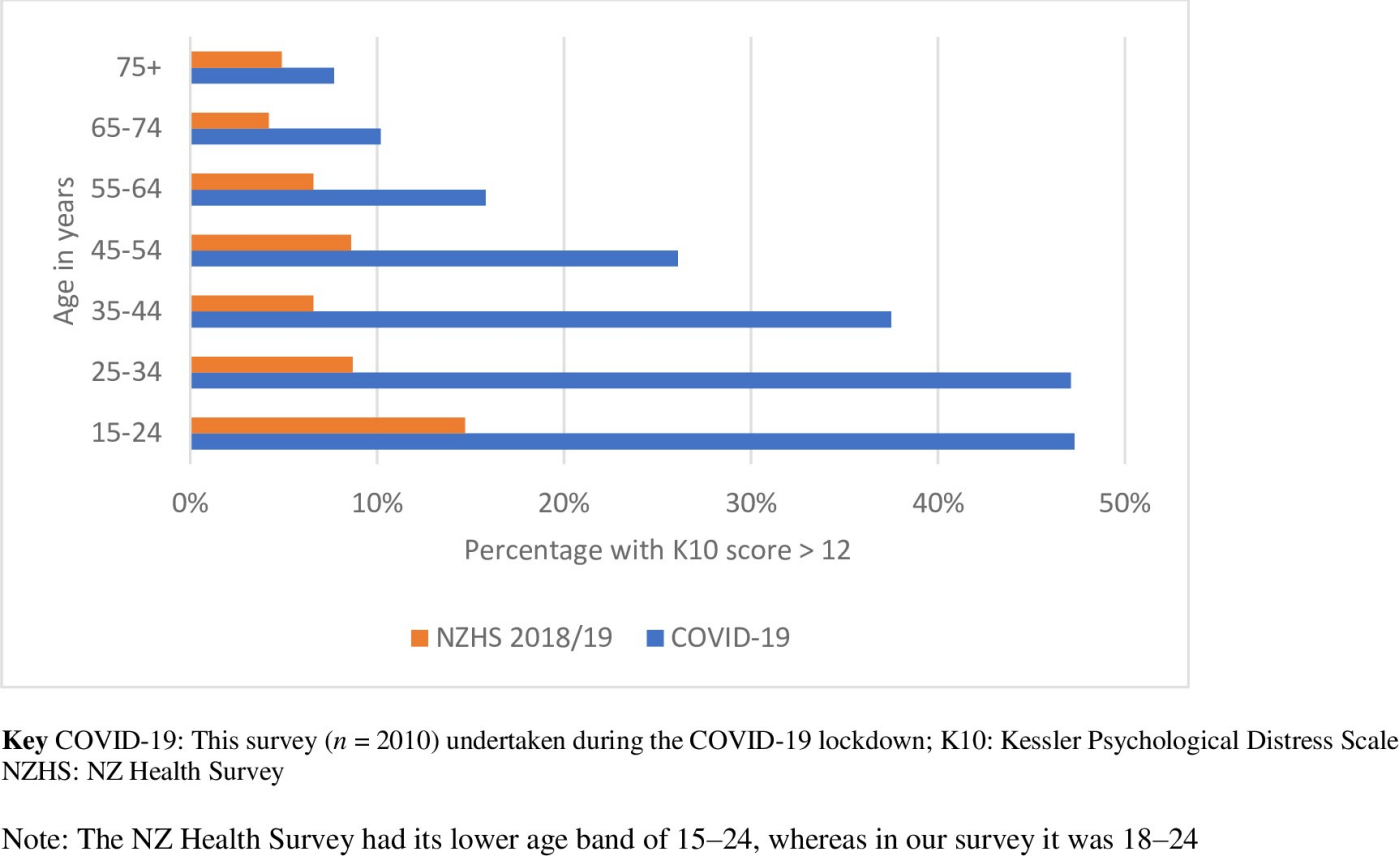
Percentage of respondents with K10 score > 12 by age in COVID-19 survey compared against baseline rates in the NZ Health Survey*.

Lockdown psychological distress analysed by age is compared with data from the NZ Health Survey in Fig 2. What is most striking in our data is the elevated level of distress in younger adults (under 44 years) compared with baseline population rates. However, it is not possible to determine the relative contribution of the COVID-19 pandemic versus the methodological differences in surveys. The NZ Health Survey is a continuous survey with probability sampling administered by face-to-face interviews, while ours was a cross-sectional demographically representative but non-random survey administered online. Incidentally, data collection for the Health Survey had to be suspended in March 2020 due to the COVID-19 lockdown restrictions, so we have no face-to-face lockdown data for comparison [27].

The baseline Health Survey data show that under normal circumstances, NZ women are around 1.8 times more likely to be experiencing psychological distress than men. Interestingly, our data collected during the lockdown showed a much smaller gender gap. In fact, the proportion of males and females experiencing moderate to severe anxiety (assessed by the GAD-7) was the same. This finding differs to studies from other countries with higher rates of community transmission than New Zealand, which report disproportionately elevated rates of anxiety and depressive symptoms in females [10, 11, 40]. One possible hypothesis is that economic anxiety (e.g., worrying about job loss, family finances), which may have dominated in NZ, affected males and females in a similar way early in the pandemic. Health-related anxieties, which may have been more evident in countries with higher infection rates, may affect females more than men [40]. Previous research has shown that being out of work may have a greater psychological toll on men than on women [41], but this is much less evident in countries where the ‘double breadwinner’ model predominates [42].

Reports of family violence seem elevated. The NZ Crime and Victims of Crimes Survey 2018/9 (Table 4) found within a 12-month period, 0.7% of adults experienced physical assault or harassment and threatening behaviour, and 0.4% experienced sexual assault by a family member [43]. In our study, the reported levels of these experiences were between three and four times higher. Again, methodological differences in data collection methods could account for some of these differences.

**Table 4:** Family harm during lockdown*.

	Lockdown (1-month prevalence) number unweighted	Lockdown (1-month prevalence) weighted % (95% CI)	NZCVCS (12-month prevalence)
**Physical assault**	42	2% (1.5, 2.7)	0.7%
**Harassment and threatening behaviour**	66	3.3% (2.4, 4.1)	0.7%
**Sexual assault**	31	1.5% (1.0, 2.0)	0.4%
**Frightened by a family member**	76	3.6% (2.8, 4.5)	Not reported

**Key** CI: confidence interval; NZCVCS; NZ Crime and Victims of Crime Survey.

Note: In total 9% of participants reported one or more forms of family harm. The percentages here do not add to 9% as some participants answered yes to multiple categories.

While the 6% of people reporting suicidal ideation also seems high, the majority described having experienced similar thoughts in the 12 months prior to the lockdown. This estimate is higher than in Te Rau Hinengaro, the NZ Mental Health survey, which gave a 3.2% prevalence of suicidal ideation over a year [44]. However, Te Rau Hinengaro data is now over 15 years old, and may no longer reflect the epidemiology of suicidal ideation in NZ. On the other hand, there may be selection bias within our survey, such that for some reason there are higher rates of suicidal ideation than in the general population. Nonetheless, that one in 16 people within our survey reported having suicidal thoughts over the lockdown period is of concern and warrants further research.

### Examination of vulnerable groups

Vulnerable groups included those with past histories of mental illness, young people, and essential workers. We had identified the elderly as a priority group *a priori* because of their higher risks of social isolation, lack of digital connectivity and greater vulnerability to COVID-19. However, similar to other research that has consistently found declining prevalence of mental disorders with increasing age [45–47], in NZ older people appeared to be faring better than younger people. This is not to say older people were unscathed. In our survey, psychological distress was more prevalent among people of all age groups when compared with prevalence in the same age bracket in the NZ Health Survey. However, the magnitude of increased prevalence was less than that observed in the younger age groups. This trend has also been observed in other international samples [e.g., 13, 48]. Finding lower prevalence of distress among older people reflects their higher baseline wellbeing and may also reflect resilience from having overcome past adversities and experiencing fewer daily disruptions and economic impacts. Older people may also have felt they were safer in NZ than elsewhere. While the virus caused clusters in five aged-care residential facilities, these were contained relatively quickly and COVID-19 did not become endemic amongst services for the elderly. To date, only 7.6% of NZ cases in have occurred in people over 70, with 19 deaths in this age range [5]. Lastly, the older people who had the technology and skills to access and complete this online survey could differ systematically from their peers without these resources.

Those with a past history of mental illness had an OR of just under 3 (2.9, 95% C 2.2–3.8) for moderate to high psychosocial distress. While this group may well have had elevated rates of distress prior to the lockdown, we note that about one in three thought their mental health was worse than usual, while only one in six reported it was better than usual.

We found essential workers showed increased rates of distress, but not to the extent of other international studies [9]. During a pandemic, essential workers in general and health professionals in particular, may face multiple stressors, including an increased risk of infection and inadequate protection from contamination, frustration, discrimination, having to rationalise limited resources, sick and dying patients, grief, a lack of contact with families, fear of infecting others, overwork and exhaustion [49]. The rates of distress in NZ essential workers were lower than in international samples presumably because fewer workers were directly exposed to COVID-19 than in countries where COVID-19 was rampant. However, it should be noted that more than one in 10 cases of COVID-19 were in healthcare workers [50].

### Comparison with international studies

While there are now a number of international studies emerging on the psychosocial impacts of COVID-19, our research is unique in that it occurred during a stringent lockdown which was part of the New Zealand Government’s COVID-19 ‘elimination strategy’ [51, 52] and which was showing positive results during the time of data collection (see Fig 1). This means we examined the impact of strict lockdown restrictions in the absence of widespread direct effects of the virus. This provides evidence on the possible psychological effects of such restrictions for future policy makers weighing their risks and benefits in pandemic response planning.

To date (August 2020), NZ has experienced 22 COVID-19 related deaths, which is the lowest COVID-19 related mortality in the OECD [53]. Nevertheless, the increased rates of mental distress during lockdown reported in our study are consistent with international evidence showing significant negative effects on population wellbeing [e.g., 7, 8, 54–57]. Essential workers, young people, those economically impacted, and those with pre-existing conditions, including mental illness, have similarly been shown to be at high risk. [9, 58, 59]. Reports from China, the US, Brazil, and Australia also indicate increases in family violence coinciding with lockdown orders [54–57].

### Limitations

Like all survey-based research our study has some limitations. Our data was generated by participants’ subjective reports of their experiences and emotions. The self reporting of depressive or anxiety symptoms is not equivalent to a structured diagnostic interview [60], and cannot be used to classify a mental disorder like a depressive disorder or, in aggregate, to estimate the prevalence of disorder based on diagnostic criteria. Our data was also collected cross sectionally and does not reveal whether the increases in psychological distress were sustained over time.

Panel samples generally tend to over-represent those with higher socio-economic status and education, and access to a computer or internet-connected mobile phone was necessary to complete the survey. Consequently, those most affected by the pandemic lockdown may well be under-represented in our sample. On the other hand, people for whom the topic of wellbeing had particular salience (perhaps because they were experiencing difficulties), may have been more inclined to participate. Hence, selection bias may have influenced our results, though the direction and magnitude of its effects are hard to quantify.

Comparisons between our results and those of other surveys are also complicated by differences in sampling frames and survey modes (e.g. web-based versus face-to-face interviews in the NZ Health Survey). However, we used sample quotas and post-stratification weighting to ensure a gender, ethnicity and age balance representative of the NZ population. The survey was peer reviewed, pre-tested on a sample of members of the general public, and modified accordingly [37]. We used validated outcome measures for which there are established baseline norms. The survey could be completed on a mobile phone, tablet or computer. Consent and completion rates were high.

### Next steps

It is clear that the consequences of the novel coronavirus pandemic will be pervasive and prolonged. Evidence of second-wave community transmission in neighbouring countries and enduring economic impacts suggest COVID-19’s effects on mental wellbeing will continue. After NZ’s early success with 100 consecutive days of no community transmission, the re-emergence of cases in Auckland on 11 August 2020 left the country wondering whether another large outbreak was imminent and what measures should be implemented to control it.

The ongoing collection of robust mental health data is imperative to guide policy and service delivery. Methods should include random population sampling, validated measures (with benchmark data), and the target sample should be adequately powered to assess critical but low prevalence outcomes such as suicide and family harm. The established methods of probability sampling have been sorely challenged by COVID-19 restrictions, but should resume as soon as they can be undertaken safely. Further research into priority groups (such as young people, those with pre-existing mental health conditions, ethnic minorities, people who are socioeconomically disadvantaged, those who lose work, and those at the front line) should occur. Qualitative research could probe the lived experiences of the lockdown for priority groups. As well as investing in research examining vulnerabilities, the protective factors for those who coped well during the crisis should also be explored.

In due course, analysing any changes in mental health-related national data over the course of the COVID-19 pandemic will give further information on its psychological impacts. Useful data to interrogate will include service utilisation data (e.g., mental health presentations in primary care, psychiatric referrals), psychotropic prescribing rates, suicide rates, and family harm data.

Governments should treat the adequate provision of psychosocial support with similar priority to other health measures. Current and future response plans need to take due account of the psychological burdens of pandemics and how to mitigate them. As an example, we note the NZ Government’s release of the Psychosocial and Mental Wellbeing Recovery Plan outlining the national approach to supporting the mental and social wellbeing in the COVID-19 recovery period [61].

Supporting the population’s psychological wellbeing spans a broad range of domains, from ensuring people have ready access to accurate information, basic necessities, and community connection at one end, through to the availability of specialist mental health services at the other end. Communication campaigns are required to publicise what supports are available and how people can access them, with targeted messages for priority groups.

The mental health sector, which even prior to the COVID-19 pandemic has been struggling under pressure in many countries including NZ [62], needs to be adequately resourced to meet the predicted increased demand. Free access to high quality e-therapies and telehealth support also becomes increasingly important if people are afraid or are not allowed to leave their homes.

## Conclusions

NZ is noteworthy for having implemented stringent restrictions early in the COVID-19 pandemic, and for eliminating the virus from its general populace between May and August 2020. A significant proportion of the population was adversely affected by the lockdown. While the economic impacts of the lockdown measures were serious and are ongoing, our study emphasises the need to pay equal attention to the effects of such lockdowns on mental wellbeing. Governments dealing with pandemics like COVID-19 should treat the adequate provision of psychosocial support with similar priority to contact tracing, provision of personal protective equipment, and procurement of ventilators.

## Supporting information

S1 File (PDF)
