# Supplementary material for: Psychological distress, anxiety, family violence, suicidality, and wellbeing in New Zealand during the COVID-19 lockdown: A cross-sectional study
Source: PLoS One. 2020 Nov 4;15(11):e0241658. doi: 10.1371/journal.pone.0241658 (PMC7641386; doi:10.1371/journal.pone.0241658)
Supplement: S1 File — (PDF) [file pone.0241658.s001.pdf]

1. Introduction

Kia ora and welcome

Thank you for clicking through to our survey; it should take you around 10 to 15 minutes to complete.

The survey is being conducted by researchers from the University of Otago, with generous support from Dynata.

To go directly to the survey please click on the 'Next' button at the bottom of the page

If you lose your connection to the Internet or this survey at any point, please click the link provided in the email you received and it will take you back to the point where you left off.

Example only

THE EFFECTS OF COVID-19 AND THE LOCKDOWN ON WELLBEING IN NEW ZEALAND

Information for participants

What will participants be asked to do? The COVID-19 pandemic has resulted in major disruptions to our lives. Research from overseas has shown that pandemics and lockdowns have significant effects on people's well-being and mental health, but we do not yet have similar research from Aotearoa New Zealand. We are interested in the experience of New Zealanders and this is what we would like to ask you about. Should you agree to take part in this survey, you will be asked about your experiences over the course of the COVID-19 lockdown, including how it has affected your employment, your mental health and well-being, your behaviours, and any 'silver linings' or positive experiences. The survey should take about 10 to 15 minutes to complete.

Thank you for showing an interest in this study. Please read this information sheet carefully before deciding whether or not to participate. If you decide to participate, we thank you. If you decide not to take part there will be no disadvantage to you and we thank you for considering our request.

What is the aim of the project? This study explores the experiences of the New Zealand population during the current COVID-19 event, including the subsequent imposed social isolation measures (the Level 4 'lockdown').

What types of participants are being sought? We are seeking 2000 adult participants from the general population aged 16 years and older. We are applying quotas so we ensure our sample has reasonable numbers of people from different ethnicities, and roughly similar numbers across genders.

What data or information will be collected and what use will be made of it? All your answers will be completely anonymous to the research team, only Dynata will know the identity of participants. The results of the project may be published and will be available in the University of Otago (New Zealand) library; however there will be no way to trace responses back to individuals therefore the anonymity of participants will be preserved. The results will also be shared with the Ministry of Health to inform the support packages they offer in response to the COVID-19 crisis. The data collected will be securely stored in such a way that only members of the research team will be able to gain access to it. Data obtained as a result of the research will be retained for at least five years in secure storage. Any information held may be destroyed after five years even though the data derived from the research will, in most cases, be kept for much longer or possibly indefinitely.

Example only

Can participants change their mind and withdraw from the project? You may withdraw from participation in the survey at any time and without any disadvantage to yourself.

What supports are available? Some of the questions are about potentially sensitive topics like suicidal thoughts or family violence. We are asking about these topics because overseas evidence has suggested changes in their frequency related to the COVID-19 crisis. Like all other questions, your response to these questions is anonymous. You do not have to answer

any question you do not wish to. If you feel negatively affected thinking about any of these topics, please use the contact details for the support services provided or free call or text 1737 any time, 24 hours a day to talk to a trained counsellor. This service is completely free.

What if participants have any questions? If you have any questions about our project, either now or in the future, please feel free to contact:

Dr Matthew Jenkins
Department of Psychological Medicine, University of Otago, Wellington
Email Address: matthew.jenkins@otago.ac.nz

This study has been approved by the University of Otago Human Ethics Committee (reference F20/003). However, if you have any concerns about the ethical conduct of the research you may contact the University of Otago Human Ethics Committee (Gary Witte: gary.witte@otago.ac.nz or Jo Farron de Diaz: jo.farrondediaz@otago.ac.nz). Any issues you raise will be treated in confidence and investigated and you will be informed of the outcome.

Example only

As part of the University of Otago's ethics approval process we need to ask you to answer the following question before starting our survey.

I have read the Information Sheet for this project and agree to take part in the study.

- ○ Yes
- ○ No

First, a few questions to see if you qualify for this survey.

Which of these do you most identify with?

- ○ Male
- ○ Female
- ○ Gender diverse

Which age group are you in?

- ○ Under 18
- ○ 18-34
- ○ 35-54
- ○ 55-74
- ○ 75 and older

Example only

Which of these ethnic groups do you identify with?

Please select ALL the ethnic groups that you identify with.

- ☐ New Zealand European
- ☐ Māori
- ☐ Samoan
- ☐ Cook Island Māori
- ☐ Tongan
- ☐ Niuean

- [ ] Chinese
- [ ] Indian
- [ ] Other (e.g. Dutch, Japanese, Tokelauan). Please state:

Do you belong to any local iwi close to where you live?

- ○ Yes
- ○ No
- ○ Don't know

Example only

Thinking about your connection to Māori culture, <u>in the last 12 months</u>, but prior to lockdown, did you do any of these things?

Please tick all that apply

- [ ] Went to a marae
- [ ] Went to a Māori festival (e.g., Pā Wars. Matariki, or Waitangi Day)
- [ ] Sang a Māori song, performed a haka, given a mihi or speech, or taken part in Māori performing arts or crafts
- [ ] Took part in traditional Māori healing or massage.
- [ ] None of these

In the last 12 months, other than those listed in the previous question, are there any other important ways that you have connected to or expressed your Māori identity or heritage.

- ○ [ ] Yes (please specify)
- ○ No
- ○ Don't know

Example only

Your living circumstances

These questions are about your living circumstances during the COVID-19 lockdown, which started on Thursday 26 March.

We define your 'bubble' as the household that you are in during the lockdown period, including anybody you are living with. Please note - this does not include people in other households, if you are living alone during lockdown.

During the lockdown, who has been living with you in your 'bubble'?

- ○ No one, I live by myself and have no pets
- ○ No one, I live by myself but I have one or more pets
- ○ Flatmates or tenants

- ○ Adult family/whānau member(s) only
- ○ Family/whānau members, including a child/children
- ○ Friends
- ○ A mixture of flatmates, family/whānau members or friends

Including yourself, how many people live with you in your bubble?

☐

Not everybody has ended up in their bubble of choice.

How satisfied are you with the bubble you are in?

- ○ Extremely dissatisfied
- ○ Dissatisfied
- ○ Neither satisfied nor dissatisfied
- ○ Satisfied
- ○ Extremely satisfied

Example only

In your lockdown residence, which of the following are available to you?

Please tick all that apply.

- [ ] Personal or quiet space
- [ ] Internet
- [ ] Computer
- [ ] Easy access to a garden/green space
- [ ] None of these

<u>During the lockdown</u>, how easy has it been to stay connected with your family/whānau and friends outside your 'bubble'?

- ○ Extremely hard
- ○ Somewhat hard
- ○ Neither easy nor hard
- ○ Somewhat easy
- ○ Extremely easy
- ○ I haven't tried to stay connected

Example only

<u>During the lockdown</u>, how often have you connected <u>each week</u> with your family/whānau and friends outside your bubble?

	Not at all	Once a week	2-3 times a week	4-6 times a week	Every day
Talked in person	○	○	○	○	○
Talked by video (eg, Skype, WhatsApp)	○	○	○	○	○

	Not at all	Once a week	2-3 times a week	4-6 times a week	Every day
Talked over the telephone	○	○	○	○	○
Connected by writing (text, email, snail mail)	○	○	○	○	○

Since the lockdown began, how has your level of contact changed with family/whānau and friends outside your bubble?

- It has increased
- It has decreased
- It has stayed the same

Example only

Overall, thinking about how well your family/whānau has been doing since the lockdown, would you say that things are currently getting better, getting worse, or staying about the same?

- Getting better
- Getting worse
- Staying the same

During the lockdown, how are you and the people you are living with getting along with each other?

- Very badly
- Badly
- Neither well nor badly
- Well
- Very well

<u>During the lockdown</u>, how often have you felt lonely or isolated?

- All of the time
- Most of the time
- Some of the time
- A little of the time
- None of the time

Example only

<u>During the lockdown</u> how much time have you been spending looking at information related to COVID-19?

- Less than an hour a day
- 1-2 hours per day
- 2-4 hours per day
- 4-8 hours per day
- More than 8 hours a day

The following questions are about jobs and businesses during the COVID-19 lockdown.

Do you have a job now?

- Yes, I have a job
- No, I don't have a job
- I am self-employed
- I am a business owner
- I am retired
- I have never had a job

Example only

Have your hours of paid work been drastically reduced as a result of the lockdown?

- Yes
- No
- Doesn't apply to me

Has your paid workload drastically increased as a result of the lockdown?

○ Yes
○ No
○ Doesn't apply to me

Have you lost your job (or jobs) as a result of the lockdown?

○ Yes
○ No
○ I didn't have a job before the lockdown

Example only

Are you an 'essential worker' (e.g., healthcare, law enforcement, emergency services, provider of essential goods)?

○ Yes
○ No

Is someone in your bubble an essential worker (e.g., healthcare, law enforcement, emergency services, provider of essential goods)?

○ Yes
○ No
○ Not applicable

What type of essential work do you do?

- Healthcare
- Law enforcement
- Other emergency services (e.g., fire service)
- Provider of other essential goods or services (e.g., food supply, fuel, waste removal, internet, financial support, transport)

Through your job, have you had known direct contact with COVID-19 patients?

- I have had direct contact with people who I knew at the time were **suspected of having** COVID-19
- I have had direct contact with people who I knew at the time had **been diagnosed with** COVID-19
- I found out later that people I had contact with were probable or confirmed COVID-19 cases but I did not know at the time
- I may have had contact with probable or confirmed COVID-19 cases
- To the best of my knowledge, I have not yet had contact with probable or confirmed COVID-19 cases

Example only

Do you personally own or run a business whose reduction in turnover due to COVID-19 threatens the survival of your business?

- Yes
- No

Have you personally had to make people in your organisation redundant or lose their jobs?

- ○ Yes
- ○ No

If there has been a reduction in your hours, have you applied (or has your employer applied on your behalf) for any of the following?

Please select all that apply.

- ☐ COVID-19 wage subsidy
- ☐ COVID-19 leave payment
- ☐ Financial support for your business
- ☐ Other government financial support
- ☐ None of the above
- ☐ Not applicable to me
- ☐ I don't know

Example only

How much has any reduction in your hours, losing your job, or loss of turnover in your business made it more difficult for you to meet basic living costs such as rent, mortgage payments, or food bills?

- ○ A great deal

- A lot
- A moderate amount
- A little
- None at all
- Not applicable to me

Your general health

The next few questions are about your general (physical) health.

Example only

How would you describe your general (physical) health?

- Poor
- Fair
- Good
- Very good
- Excellent

Over the past 5 years, have you had a medical condition that may make you more vulnerable to COVID-19 such as heart disease, COPD (difficulty breathing), weakened

immunity, or cancer?

- Yes
- No
- Prefer not to say

Do you have a family member who has a medical condition that may make them more vulnerable to COVID-19 such as heart disease, COPD (difficulty breathing), weakened immunity, or cancer?

- Yes
- No
- Prefer not to say

Example only

Do you live with somebody, apart from a family member, who has a medical condition that may make them more vulnerable to COVID-19 such as heart disease, COPD (difficulty breathing), weakened immunity, or cancer?

- Yes
- No
- Prefer not to say

Do you think you have had COVID-19?

- Yes
- No
- Not sure

Have you been tested for COVID-19?

- Yes
- No

What were the results of this test?

- Positive
- Negative
- Awaiting results

Example only

Have you fully recovered from COVID-19?

- Yes
- No

Do you have any physical condition or disability that affects your ability to function (e.g., leave the house for essential goods or for physical activity) during the lockdown?

○ Yes

○ No

Alcohol and Smoking

The following questions are about your alcohol intake and smoking since the start of the COVID-19 lockdown.

Example only

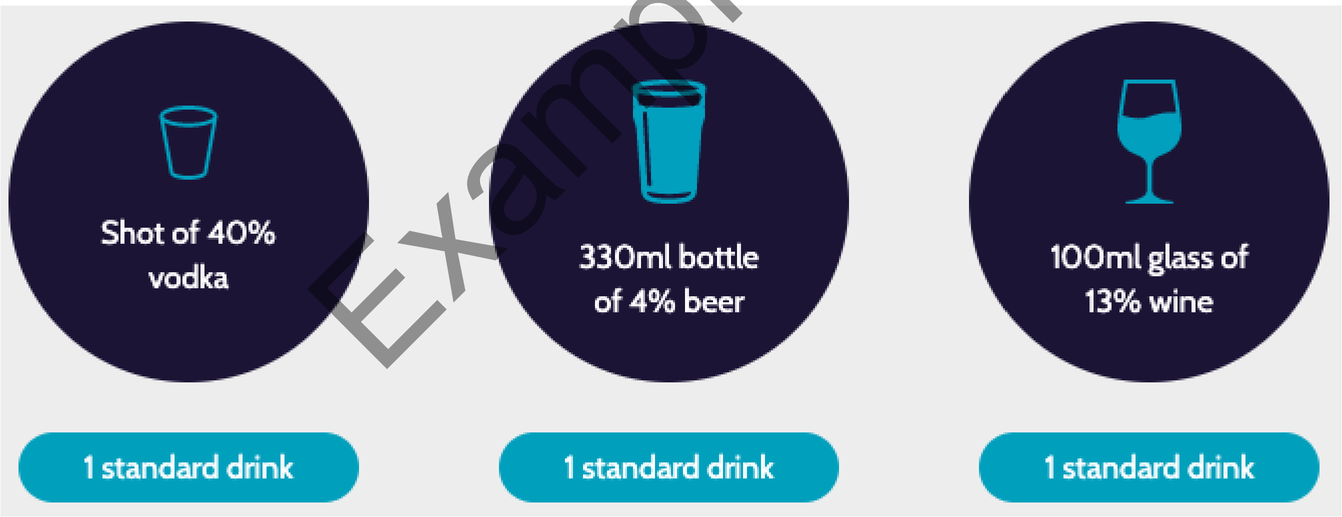

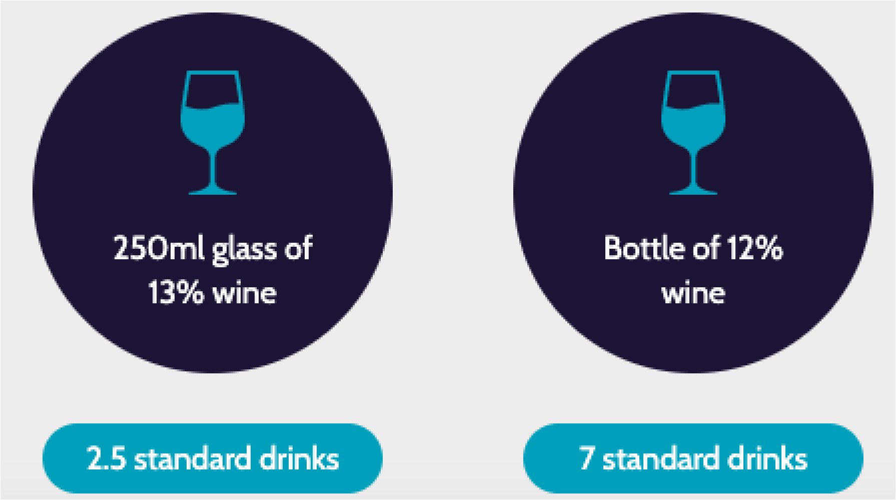

Using the above graphic as a guide, BEFORE the lockdown, how many standard drinks would you have consumed in a typical 7 days?

Please answer using a number

Example only

How many standard drinks have you consumed in the last 7 days?

Please answer using a number

How often do you CURRENTLY smoke tobacco cigarettes (either tailor-made or roll-your-own)?

- I smoke cigarettes every day
- I smoke cigarettes at least once a week, but not daily
- I smoke cigarettes less than once a week
- I am an ex-smoker
- I have never been a smoker

Example only

BEFORE the lockdown, about how many cigarettes did you smoke each day?

Please answer using a number

DURING the lockdown, about how many cigarettes do you smoke each day?

Please answer using a number

BEFORE the lockdown, about how many cigarettes did you smoke each week?

Please answer using a number

DURING the lockdown, about how many cigarettes do you smoke each week?

Please answer using a number

Example nly

How are you feeling?

The next questions are about your mental health and wellbeing. We are interested in whether people's mental health or wellbeing may have been affected by recent COVID-19 events.

Some of these questions may seem a bit repetitive but they come from surveys that are used all over the world. Please bear with us and answer them all.

Like all of the questions in the survey, your answers are completely confidential and anonymous, and will be used for research purposes only.

DURING the lockdown:

	At no time	Some of the time	Less than half of the time	More than half of the time	Most of the time	All of the time
I have felt cheerful and in good spirits	○	○	○	○	○	○
I have felt calm and relaxed	○	○	○	○	○	○
I have felt active and vigorous	○	○	○	○	○	○
I woke up feeling fresh and rested	○	○	○	○	○	○
My daily life has filled me with things that interest me	○	○	○	○	○	○

Example only

The following question refers to your overall sleep quality for most nights both before and since the lockdown.

Please think about the quality of your sleep overall, such as how many hours of sleep you got, how easily you fell asleep, how often you woke up during the night (except to go to the bathroom), how often you woke up earlier than you had to in the morning, and how refreshing your sleep was.

<u>BEFORE the lockdown</u>, how would you rate your sleep quality overall?

1 (Terrible)	2	3	4	5	6	7	8	9	10 (Excellent)
○	○	○	○	○	○	○	○	○	○

<u>DURING the lockdown</u>, how would you rate your sleep quality overall?

1 (Terrible)	2	3	4	5	6	7	8	9	10 (Excellent)
○	○	○	○	○	○	○	○	○	○

Example only

<u>DURING the lockdown</u>, how often have you been bothered by the following?

	Not at all	Some days	Most days	Nearly every day
Feeling nervous, anxious, or on edge	○	○	○	○
Not being able to stop or control worrying	○	○	○	○
Worrying too much about different things	○	○	○	○
Having trouble relaxing	○	○	○	○
Being so restless that it's hard to sit still	○	○	○	○
Becoming easily annoyed or irritable	○	○	○	○

	Not at all	Some days	Most days	Nearly every day
Feeling afraid as if something awful might happen	○	○	○	○

DURING the lockdown, about how often have you felt tired out for no good reason?

- ○ None of the time
- ○ A little of the time
- ○ Some of the time
- ○ Most of the time
- ○ All of the time
- ○ Don't know

Example only

DURING the lockdown, about how often have you felt nervous?

- ○ None of the time
- ○ A little of the time
- ○ Some of the time
- ○ Most of the time
- ○ All of the time
- ○ Don't know

DURING the lockdown about how often have you felt so nervous that nothing could calm you down?

- None of the time
- A little of the time
- Some of the time
- Most of the time
- All of the time
- Don't know

Example only

DURING the lockdown, about how often have you felt hopeless?

- None of the time
- A little of the time
- Some of the time
- Most of the time
- All of the time
- Don't know

DURING the lockdown, about how often have you felt restless or fidgety?

- None of the time
- A little of the time
- Some of the time
- Most of the time

- All of the time
- Don't know

DURING the lockdown, about how often have you felt so restless you could not sit still?

- None of the time
- A little of the time
- Some of the time
- Most of the time
- All of the time
- Don't know

Example only

DURING the lockdown, about how often have you felt depressed?

- None of the time
- A little of the time
- Some of the time
- Most of the time
- All of the time
- Don't know

DURING the lockdown, about how often have you felt that everything was an effort?

- None of the time
- A little of the time
- Some of the time
- Most of the time
- All of the time
- Don't know

DURING the lockdown, about how often have you felt so sad that nothing could cheer you up?

- None of the time
- A little of the time
- Some of the time
- Most of the time
- All of the time
- Don't know

Example only

DURING the lockdown about how often have you felt worthless?

- None of the time
- A little of the time
- Some of the time
- Most of the time
- All of the time
- Don't know

DURING the lockdown what have you found to be the main sources of stress or anxiety for you?

Please tick all that apply

- ☐ Uncertainty regarding my health
- ☐ Uncertainty regarding the health of my family or friends
- ☐ Uncertainty regarding my finances
- ☐ Uncertainty regarding my employment security
- ☐ The wider consequences of COVID-19
- ☐ Not applicable
- ☐ [ ] Something else

Example only

Have you previously been diagnosed with a mental illness by a doctor or psychologist?

- ○ Yes
- ○ No
- ○ Prefer not to say

What diagnosis or diagnoses did they make?

Please tick all that apply

- ☐ Depression
- ☐ Bipolar disorder
- ☐ Anxiety disorder
- ☐ Personality disorder
- ☐ Psychotic disorder
- ☐ Alcohol or drug disorder
- ☐ Other
- ☐ Don't know
- ☐ Prefer not to say

DURING the lockdown, how is your mental health compared to usual?

- ○ Much worse than usual
- ○ Worse than usual
- ○ The same as usual
- ○ Better than usual
- ○ Much better than usual
- ○ Prefer not to say

Example only

Important

If any of these questions have caused you to feel distressed, or if you are struggling with your mental health, please free call or text 1737 or visit https://1737.org.nz to speak to a trained counseller. This also applies if you have any concerns for your friends, family or whānau.

Alternatively, you can call Depression.org.nz on 0800 111 757 or text 4202.

Have you ever been exposed to any of the following (aside from the current COVID-related events)?

Please tick all that apply

- ☐ Childhood adversity (neglect, physical or sexual abuse)
- ☐ Physical or sexual abuse after the age of 16
- ☐ Exposure to a traumatic event involving physical or sexual abuse to others
- ☐ Natural disaster (e.g., fire, flood, earthquake)
- ☐ Serious physical injury (e.g. car accident)
- ☐ Serious illness
- ☐ Other (please state) ______
- ☐ None of the above

Example only

Please note: The following questions ask about potentially sensitive topics.

Like all of the questions in this survey, your answers are completely confidential and anonymous, and will be used for research purposes only.

But, if there are some questions you would prefer not to answer, just skip them.

BEFORE the lockdown, but during the previous 12 months, had you:

	Yes	No	Prefer not to say
**Seriously thought about ending your own life?**	◯	◯	◯
**Made plans to end your own life?**	◯	◯	◯
**Made an attempt to end your own life?**	◯	◯	◯

Example only

DURING the lockdown, have you:

	Yes	No	Prefer not to say
**Seriously thought about ending your own life?**	◯	◯	◯
**Made plans to end your own life?**	◯	◯	◯

	Yes	No	Prefer not to say
**Made an attempt to end your own life?**	○	○	○

If you feel distressed or feel like you want to talk about anything related to these issues, please call Lifeline on 0800 543 354 or text 4357.

Alternatively, visit the Lifeline website at https://www.lifeline.org.nz/.

Example only

Family violence

The next two questions are about any incidents of family violence that have occurred in your household.

Remember, you are not obliged to answer these questions, but all responses are completely confidential and anonymous, and your responses will be used for research purposes only.

If family violence is curently an issue for your family/whānau or friends, please contact one of the following organisations for assistance:

Women's Refuge crisis line on 0800 733 843 - (24 hours)

Family violence information line on 0800 456 450

Emergency services on 111.

<u>DURING</u> the lockdown, have you experienced any of the following as a result of an action from a <u>family/whānau member</u>?

Please tick all that apply, and include threats made to you directly (face-to-face, phone, email, text), or via someone else.

Example only

- ☐ Been frightened
- ☐ Been insulted or abused
- ☐ Been threatened with harm to you, your children or your pets
- ☐ Been threatened with being hit, slapped or punched
- ☐ Been threatened with a weapon or other object
- ☐ Been slapped, punched or kicked
- ☐ Been hit with a weapon or other object
- ☐ Been touched sexually in a way you didn't like
- ☐ Been forced to have sex when you didn't want to
- ☐ None of these
- ☐ Prefer not to say

<u>DURING</u> the lockdown, have you been a witness to any of the above in your 'bubble' ?

- ○ Yes

○ No

○ Prefer not to say

Useful contacts for family violence-related incidents

If family violence is currently an issue for your family/whānau or friends please contact one of the following organisations for assistance:

Women’s Refuge

Call the crisis line on 0800 733 843 (24 hours)

'It's Not OK' family violence prevention

Call the information line on 0800 456 450

Rape Crisis

Call 0800 88 33 00 or visit http://www.rapecrisisnz.org.nz/

If you are presently in danger call the emergency services on 111.

Example only

Positive aspects of COVID-19

Have you experienced any 'silver linings' or positive aspects during the COVID-19 lockdown?

Please tick all that apply

- ☐ Yes, for me personally
- ☐ Yes, for wider society
- ☐ No

What are these silver linings, for you personally or for wider society?

Example only

Finally, a few questions about yourself

Which of the following best describes your highest formal qualification?

- ○ No formal qualification
- ○ High school qualifications (school certificate, NCEA, UE, Bursary)
- ○ Certificate or diploma below Bachelor's level
- ○ Bachelor's degree
- ○ Post-graduate or higher qualification

What is your exact age (in years)?

[ ]

Which gender do you identify with?

- Male
- Female
- Gender diverse

Are you?

- Transgender female to male
- Transgender male to female
- Intersexed
- Gender non-conforming
- Genderqueer
- Two-spirit
- Third gender
- [ ] Other

Example only

Are you pregnant?

- Yes
- No
- Don't know

What is the total income that you yourself received from all sources, before tax or any other deductions, over the last 12 months?

- Less than $10,000
- $10,001 – $20,000
- $20,001 – $30,000
- $30,001 – $40,000
- $40,001 – $50,000
- $50,001 – $60,000
- $60,001 – $70,000
- $70,001 – $100,000
- $100,001 – $150,000
- $150,001 or more
- Prefer not to say

Example only

What is your postcode?

Are there any comments you'd like to make about COVID-19, the lockdown or this survey? If so, please write them in the box below.

Example only

Remember

If you are feeling distressed by any of the content in this survey, think that these issues may be affecting family/whānau members or friends, or if you simply want more information, please note the following helplines and services.

Family violence

Women's Refuge

Call the crisis line on 0800 733 843 (24 hours)

'It's Not OK' family violence prevention

Call the information line on 0800 456 450

Rape Crisis

Call 0800 88 33 00 or visit http://www.rapecrisisnz.org.nz/

If you are in danger, call the emergency services on 111.

Depression or suicide

Lifeline

Call 0800 543 354 or text 4357 or on the web at https://www.lifeline.org.nz/.

NZ free and confidential counselling

Call or text 1737 or visit https://1737.org.nz/

Depression.org.nz

Call 0800 111 757, text 4202, or visit depression.org.nz.

COVID-19 information

Call the Ministry of Health Healthline on 0800 611 116 for advice, or visit https://covid19.govt.nz/ for up-to-date and accurate information on COVID-19.

Example only

Thank you for taking part in our survey.

Please click 'Next' to submit your answers.

Powered by Qualtrics

Example only
